# Regulation of reactive oxygen and nitrogen species by salicylic acid in rice plants under salinity stress conditions

**DOI:** 10.1371/journal.pone.0192650

**Published:** 2018-03-20

**Authors:** Yoonha Kim, Bong-Gyu Mun, Abdul Latif Khan, Muhammad Waqas, Hyun-Ho Kim, Raheem Shahzad, Muhammad Imran, Byung-Wook Yun, In-Jung Lee

**Affiliations:** 1 Division of Plant Biosciences, Kyungpook National University, Daegu, South Korea; 2 UoN Chair of Oman's Medicinal Plants & Marine Natural Products, University of Nizwa, Nizwa, Oman; 3 Department of Agriculture Extension, Government of Khyber Pakhtunkhwa, Buner, Pakistan; National Taiwan University, TAIWAN

## Abstract

This study investigated the regulatory role of exogenous salicylic acid (SA) in rice and its effects on toxic reactive oxygen and nitrogen species during short-term salinity stress. SA application (0.5 and 1.0 mM) during salinity-induced stress (100 mM NaCl) resulted in significantly longer shoot length and higher chlorophyll and biomass accumulation than with salinity stress alone. NaCl-induced reactive oxygen species production led to increased levels of lipid peroxidation in rice plants, which were significantly reduced following SA application. A similar finding was observed for superoxide dismutase; however, catalase (CAT) and ascorbate peroxidase (APX) were significantly reduced in rice plants treated with SA and NaCl alone and in combination. The relative mRNA expression of *OsCATA* and *OsAPX1* was lower in rice plants during SA stress. Regarding nitrogenous species, *S*-nitrosothiol (SNO) was significantly reduced initially (one day after treatment [DAT]) but then increased in plants subjected to single or combined stress conditions. Genes related to SNO biosynthesis, *S*-*nitrosoglutathione reductase* (*GSNOR1*), *NO synthase-like activity* (*NOA*), and *nitrite reductase (NIR*) were also assessed. The mRNA expression of *GSNOR1* was increased relative to that of the control, whereas *OsNOA* was expressed at higher levels in plants treated with SA and NaCl alone relative to the control. The mRNA expression of *OsNR* was decreased in plants subjected to single or combination treatment, except at 2 DAT, compared to the control. In conclusion, the current findings suggest that SA can regulate the generation of NaCl-induced oxygen and nitrogen reactive species in rice plants.

## Introduction

High salinity is caused by improper irrigation and drainage and affects over half of the productive irrigated land globally at an average rate of up to one-half million hectares per year [1]. Rice paddy fields also experience increased salinity due to water evaporation during the summer season, which reduces crop growth and yield [2]. Rice plants—which provide food for over 70% of the human population—are exposed to salinity stress, and the concurrent generation of reactive oxygen species (ROS) also accelerates cell damage. During abiotic stress, plant cells are exposed to an array of ROS, such as superoxide radicals (O_2_^−^), hydrogen peroxide (H_2_O_2_), hydroxyl radicals (OH^−^), and singlet oxygen (^1^O_2_), during the photosynthetic processes of photosystems I and II [3–5]. ROS generation maintains stable growth during non-stress conditions; however, under abiotic stresses, internal ROS activity increases rapidly, subsequently attacking biomolecules such as lipids, proteins, and nucleic acids [6–8]. Thus, to protect cellular components, plants must recruit antioxidant enzymes such as ascorbate peroxidase (APX), catalase (CAT), glutathione (GSH), and superoxide dismutase (SOD) [8, 9], which act to eliminate oxygenated radicals [10]. The stress signaling molecule nitric oxide (NO) is a highly reactive free radical, which may substantially participate in plant development and immunity [11]. NO is highly toxic to plants in the presence of ROS due to its chemical properties; thus, it must be converted by the plants into a non-toxic form, such as S-nitrosoglutathione (GSNO) [12, 13]. Furthermore, NO can covalently bind to cysteine residues through S-nitrosylation, and, thus, NO can be converted to nitrosylated proteins (SNOs). Although the importance of NO in plants has been recognized in various studies, the exact NO signaling pathway remains unknown [12–15].

During stress conditions, the plant hormones jasmonic acid (JA), salicylic acid (SA), and ethylene are produced as part of the stress response. In particular, SA is regarded as a key hormone involved in plant systematic acquired resistance [9]. In dicot plants such as *Nicotiana tabacum* and *Arabidopsis* spp., SA exists at low levels (approximately 50 ng·g^−1^ fresh weight), whereas in others, such as rice (*Oryza* spp.), the SA content is higher under normal growth conditions [16]. Under normal growth conditions, SA can regulate various physiological and biochemical responses, including seed germination [17–18], photosynthesis pigment content [19–20], transpiration rate, stomatal conductance, internal CO_2_ concentration [4, 21–22], nitrate reductase activity [6], endogenous hormones [4, 17], and antioxidant activities [6, 23–24]. Thus, SA application triggers biotic or abiotic stress tolerance in plants through the regulation of these physiological or biochemical mechanisms [24]. SA induced systemic acquired resistance in response to phytopathogens attack. Upon infection, when the SA pathway is activated at the site of infection, the defense response is often activated in distal plant parts to protect undamaged tissues [25]. In addition, SA plays a role in the mitigation of abiotic stresses, such as toxic metals, heat, drought, and ultraviolet radiation stresses in plants, via the regulation of ions, antioxidant enzymes, endogenous hormones, and SA synthesis-related gene expression [25–29]. Particularly, accumulated SA in plants can induce saline tolerance by initiating a cascade of endogenous hormone signaling pathways. Conversely, findings suggest that high SA accumulation causes an oxidative burst and induces programmed cell death (PCD) in *Arabidopsis* under ozone stress [25]. García-Heredia et al. [30] demonstrated that acetylsalicylic acid (ASA) and SA application induced PCD in *Arabidopsis* cell cultures via enhanced H_2_O_2_ production, and thus, cell membranes were subjected to lipid peroxidation via oxidative damage.

Plants face multiple abiotic and biotic stress conditions. For example, elevated temperature or rainfall significantly influence the spread of pests and pathogens, thus inducing enormous yields [31]. To prevent crop yield loss, there is a need for studies investigating multiple stresses. Moreover, the role of exogenous SA application and its physiological response in plants under biotic stress is well reported. However, under abiotic stress—particularly salinity stress—it has not been fully explored. Likewise, the responses of ROS and reactive nitrogen species (RNS) during multi stress conditions remain unexplored. Therefore, the current study was undertaken to examine the response of rice in exogenous SA application under salinity stress, particularly to investigate the scavengers or ROS and RNS regulation. In addition, the expression of genes related to the regulation of ROS and RNS were also examined.

## Material and methods

### Plant material and experimental design

Rice seeds (*Oryza sativa* L. ‘Dongjin’), donated by the National Institute of Crop Science, Rural Development Administration, Republic of Korea, were used in this study. Seeds were thoroughly sterilized with 5% sodium hypochlorite for 15 min and then washed several times with double distilled water (DDW) to remove chemicals. Rice seeds were germinated in an incubator (room temperature25°C) for 3 days and then sown on autoclaved sand medium for 7 days. For consistency, we selected seedlings of similar lengths for transplant. Transplanted rice seedlings were cultivated in a hydroponic medium, Yoshida solution [32], for 1 week in the growth chamber. Two days following transplantation, Yoshida solution in designated pots was supplemented with 0.5 or 1.0 mM SA, or 100 mM NaCl alone, or either 0.5 or 1.0 mM SA in combination with 100 mM NaCl. Nineteen-day-old rice seedlings were divided into the following treatment groups: (i) control (growth medium), (ii) 0.5 mM SA (SA ≥ 99%, Sigma-Aldrich, USA), (iii) 1.0 mM SA, (iv) 0.5 mM SA + 100 mM NaCl (NaCl ≥ 99%, Sigma-Aldrich, USA), (v) 1.0 mM SA + 100 mM NaCl, and (vi) 100 mM NaCl. Treatments were conducted for 1, 2, 3, and 4 days. According to Munns and Tester [33], saline soil was defined as soil containing a high concentration of soluble salts, i.e. > 40 mM NaCl. Thus, we used 100 mM of NaCl to induce artificial abiotic stress (salinity stress). We previously showed that the application of 0.5 and 1.0 mM SA induces leaf wilting in rice. Similarly, the application of 1 mM SA has been reported to induce plant cell death in tomato [34]. Based on previous findings, we assumed that 100 mM NaCl was sufficient to induce abiotic stress, and both concentrations of SA were able to induce biotic stress. During the period in the growth chamber (KGC-175 VH, KOENCON, South Korea), environmental conditions were adjusted to 14 h of light (08:00–22:00, 30°C, relative humidity 70%) and 10 h of dark (22:00–08:00, 25°C, relative humidity 70%). The pH (5.1 ± 0.1) of the rice growth medium was maintained with the addition of HCl or NaOH to provide consistent growth conditions. All experiments were replicated three times.

### Determination of sodium content

Sodium content in the shoots was analyzed. Fresh shoot samples were dried at 70°C in an oven for 2 days, and then samples were ground into a very fine powder. Ground samples were treated with 3 ml of H_2_O_2_ and 5 ml of HNO_3_, decomposed by microwaving, and digested with 2–3% HNO_3_. Sodium content in rice leaves was measured by inductively-coupled plasma mass spectrometry (ICP-MS), as described by Jin and Zhu [35].

### Quantification of the endogenous hormone SA

To assess SA accumulation in rice plants, we analyzed free SA content using a modified protocol [36]. Leaf samples were collected and placed directly into liquid nitrogen. Collected samples were stored in a deep-freezer (-70°C) until further experimentation. Frozen samples were freeze-dried and ground into a very fine powder. A sample of freeze-dried leaf (0.2 g) was extracted with 90 and 100% methanol and then centrifuged at 10,000 × *g* for 20 min. Both methanol extracts were dried with a vacuum centrifuge, and dry pellets were re-dissolved in 2.5 ml 5% trichloroacetic acid. The supernatant was vortexed with ethyl acetate/cyclopentane/isopropanol (49.5:49.5:1, v/v), and the top organic layer was transferred into a 4-ml vial and then dried with purified nitrogen gas. The SA was analyzed by HPLC, which included fluorescence detection. More detailed information is listed in S1 Table.

### Analysis of electrical conductivity and chlorophyll fluorescence

To investigate electrolytic leakage from cellular tissues, electrical conductivity (EC) was measured in rice leaves. Plant leaf samples were collected at 1-day intervals, cut into 1-cm segments, and kept in 10 ml DDW for 24 h in an incubator (around 25°C). After 24 h, 0.5 ml of solution was placed on the EC meter (B-771, HORIVA Scientific, Japan) to measure EC. The remaining 9.5 ml solution was boiled at 100°C for 1 h to thoroughly elute all ions in the leaf cells, and the EC of the boiled solution was again measured. EC data were used to calculate a ratio of the values obtained from the incubated and boiled samples.

Chlorophyll fluorescence (CF) was measured using a chlorophyll fluorometer (FIM 1500, ADC Bioscientific Ltd., UK). To ensure the uniform collection of data, we used one-third of a leaf for the measurement. Collected data were used to calculate the maximum quantum yield of photosystem II (Fv/Fm), as described by Gentry et al. [37].

### Determination of hydrogen peroxide (H_2_O_2_) content

The method used to extract and quantify H_2_O_2_ has been described by Cheeseman [38]. Rice plants (shoots) were homogenized in an extraction medium containing 0.1 M K-phosphate (pH 6.4) and 5 mM KCN. The H_2_O_2_ content was measured as described by Cheeseman [38]. The assay mixture contained 250 μM ferrous ammonium sulfate, 100 μM sorbitol, and 100 μM xylenol orange in 25 mM H_2_SO_4_. The assay was modified to include 1% ethanol (EtOH), after which eFOX was used to enhance sensitivity. The H_2_O_2_ contents were measured to determine the difference in absorbance between 550–800 nm. H_2_O_2_ at 30% (dilution of reagent grade) was used as a standard.

### Determination of lipid peroxidation activity

Lipid peroxidation activity was determined using the method described by Ohkawa et al. [39]. A 100-mg fresh leaf sample was subjected to extraction using 10 mM phosphate (pH 7.0), and 0.2 ml of the homogenate was mixed with 0.2 ml 8.1% sodium dodecyl sulfate, 1.5 ml 20% acetic acid (pH 3.5), and 1.5 ml 0.81% thiobarbituric acid aqueous solution in a reaction tube. The mixture was heated in boiling water for 1 h and then cooled to room temperature. A 0.5 ml butanol/pyridine (15:1 v/v) solution was added to the mixture, which was then separated into two layers by vortexing. The lower layer was removed, and the upper organic layer was measured at 532 nm with a spectrophotometer. Tetramethoxypropane was used as an external standard, and the level of lipid peroxidase was expressed as micromoles of malondialdehyde (MDA) formed per milligram of protein.

### Determination of antioxidant activity

To measure stress levels, fresh leaf samples were used to determine CAT, SOD, and APX activity. A sample of fresh leaves (100 mg) was homogenized in a buffer containing 50 mM Tris HCl (pH 7.0), 3 mM MgCl_2_, 1 mM EDTA, and 1.0% PVP. The sample was then centrifuged at 10,000 rpm for 15 min at 4°C. The homogenate was centrifuged at 5000 × *g* at 4°C for 15 min. The total protein content was quantified using the Bradford assay [40]. Antioxidant activity was expressed as unit per milligram of protein. CAT was analyzed following the method described by Aebi, [41]. Crude enzyme extract was added to 0.5 ml of 0.2 M H_2_O_2_ in 10 mM phosphate buffer (pH 7.0). CAT activity was calculated by the decrease in H_2_O_2_ absorbance at 240 nm, and was thus defined as micrograms of H_2_O_2_ released per milligram of protein per minute.

SOD activity was assayed using the protocol described by Marklund and Marklund [42]. A fresh leaf sample (100 mg) was homogenized with 0.01 M phosphate buffer (pH 7.0) and centrifuged at 17,000 rpm for 15 min at 4°C. After centrifuging, the supernatant was removed and used as a crude enzyme extract. We prepared the reaction mixture, which included 2 ml of Tris–HCl buffer (pH 8.2), 0.5 ml 2 mM pyrogallol, and 2 ml distilled water. This was mixed with 2 ml of Tris–HCl buffer pH 8.2, 1.5 ml distilled water, 0.5 ml supernatant, and 0.5 ml 2 mM pyrogallol. The prepared assay mixture was immediately analyzed at 470 nm against a blank (which did not contain tissue homogenate or pyrogallol) at 3 min intervals in a spectrophotometer. The oxidation data for pyrogallol were taken each min for 3 min and used to define 100% auto-oxidation. The measured data were expressed as units per milligram of protein (1 unit was the amount of enzyme utilized to inhibit 50% of pyrogallol auto-oxidation per minute).

APX activity was measured using the method described by Nakano and Asada [43]. Enzyme or hydrogen peroxide was added to the reaction mixture, which consisted of 50 mM potassium phosphate pH 7.0, 0.5 mM ascorbate, 0.1 mM hydrogen peroxide, and 0.1 mM EDTA, and the decrease in absorbance was measured from 10 to 30 s at 290 nm using a spectrophotometer. The collected data were used to define the reaction rate for H_2_O_2_ independent of ascorbate oxidation.

### Determination of SNO contents and NO-related gene expression

We followed the protocol described by Yun et al. [15] for SNO analysis. Plant tissue (100 mg) was ground using a mortar and pestle with liquid nitrogen to produce a very fine powder. The powder was mixed with 1 ml extraction buffer (1 × PBS, pH 7.4) and centrifuged at 12,000 rpm for 10 min. The supernatant was placed in a fresh tube and further centrifuged at 13,000 rpm for 10 min. To quantify the proteins, 1.5 ml of dye reagent was added to 30 μl of extracted protein, and the assay was performed using the Pierce Coomassie Protein Assay Kit (Thermo Fisher Scientific, USA). All samples were measured with a spectrophotometer at 595 nm following incubation for 10 min at room temperature. For SNO determination, 100 μl of the plant extracts was injected into the reaction vessel of the Sievers’ Nitric Oxide Analyzer (NOA 280i, GE Water & Process Technologies, Ratingen, Germany) containing the reducing agent, including CuCl/cysteine with water.

### Quantitative real-time PCR

To investigate changes in gene mRNA expression among treatments, total RNA was isolated from fresh rice leaf tissue using TRIzol (Invitrogen, USA). Briefly, fresh rice leaf tissue was finely ground using liquid nitrogen, and 1 ml of TRIzol was added immediately after. Samples were then centrifuged for 5 min at 13,000 rpm and 4°C. The supernatant was transferred to a new 1.7 ml tube, and chloroform and isopropanol were added for phase separation and RNA precipitation, respectively. Centrifuge steps were conducted in between the aforementioned additions. Isolated RNA pellets were washed with 75% DEPC EtOH, dissolved in RNase-free water and treated with DNaseI. Total RNA was used for cDNA synthesis following the manufacturer’s protocol (cDNA synthesis kit, Phile, Korea). The cDNA was used as a template for real-time PCR (Eco™ Real-Time PCR, Illumina, USA). During the real-time PCR process, 2× Quantispeed SYBR Mix (PhileKoea) was used as the reaction mixture, and PCR was conducted according to the manufacturer’s protocol. *OsUBI* was used as the reference gene for data normalization, and all data were replicated three times. Detailed primer information for the real-time PCR process is listed in S2 Table.

### Statistical analysis

The experiment was repeated three times using the same external environment and treatments. Each replicate consisted of 10 plants. The data collected from each repetition were pooled and analyzed statistically. An analysis of variance (ANOVA) was used, and data were subjected to the Duncan multiple range test (DMRT) for the evaluation of significance among treatments. Pearson’s correlation coefficient (r) was also conducted to measure the strength of the association between variables. SAS 9.1 and SigmaPlot software were used for statistical analyses and graph sketching.

## Results

### Plasticity of phenotypic variation in rice plants during SA and NaCl stress

The effects of single and combined treatments of abiotic (NaCl; 100 mM) and artificial biotic (SA; 0.5 and 1.0 mM) stress inducers on rice shoot elongation and biomass are shown in Table 1. Plant growth attributes were recorded daily (1–4 days after treatment, DAT). The results revealed that SA application significantly reduced shoot length (18%) and rice plant biomass (57.1%) with or without the induction of salinity stress (Table 1). These growth attributes were significantly lower than those observed in the DW-applied control and NaCl-treated control plants. Increasing concentrations of SA created a stressful situation for plants, which progressively reduced their shoot length at 1–4 DAT (Table 1). SA, with or without NaCl, induced stress on the shoots and biomass; particularly, SA in combination with NaCl had more adverse effects on plants than NaCl alone from 1–4 DAT. The results showed that the increase in SA concentration had a further negative effect on plant growth. Results of the ANOVA suggested that there were only significant differences in shoot length between treatments; however, there were significant differences in biomass among treatments and between treatment periods. Consequently, our data indicated that shoot length and fresh weight of rice plants were significantly affected by single and multi-stress conditions. Therefore, our results show that application of different concentrations of SA, SA, and NaCl in combination, or NaCl only could induce physiological changes in rice plants.

**Table 1:** Shoot length and fresh weight of rice seedlings in response to SA application (0.5 and 1.0 mM) in the presence and absence of salt stress (100 mM).

Treatments	Shoot length (cm/plant)				Fresh weight (g/plant)			
	1DAT	2DAT	3DAT	4DAT	1DAT	2DAT	3DAT	4DAT
Control	29.4 ± 0.7a	29.9 ± 0.4a	29.9 ± 0.8a	31.9 ± 0.6a	0.85 ± 0.03a	0.94 ± 0.08a	1.36 ± 0.13a	1.68 ± 0.07a
0.5 mM SA	27.6 ± 1.5a	27.6 ± 0.4a	27.0 ± 1.1a	28.1 ± 0.5b	0.58 ± 0.12c	0.75 ± 0.03ab	0.69 ± 0.11c	0.85 ± 0.04bc
1.0 mM SA	28.9 ± 0.9a	27.3 ± 0.3a	28.6 ± 0.2a	27.1 ± 0.3b	0.80 ± 0.06ab	0.72 ± 0.03b	0.74 ± 0.07c	0.72 ± 0.06c
0.5 mM SA +100 mM NaCl	29.3 ± 0.6a	29.1 ± 1.4a	27.5 ± 1.6a	27.9 ± 1.0b	0.60 ± 0.05bc	0.67 ± 0.08b	0.63 ± 0.07c	0.73 ± 0.03c
1.0 mM SA +100 mM NaCl	29.8 ± 0.1a	27.3 ± 0.9a	29.3 ± 0.1a	28.7 ± 0.6b	0.78 ± 0.00abc	0.66 ± 0.08b	0.68 ± 0.04c	0.78 ± 0.05c
100 mM NaCl	27.9 ± 0.9a	28.9 ± 0.8a	28.5 ± 0.8a	28.4 ± 0.5b	0.74 ± 0.05abc	0.82 ± 0.09ab	1.07 ± 0.09b	0.94 ± 0.05b
ANOVA								
Treatment Periods	ns				***			
Treatments	***				***			
Replication	ns				ns			
Treatment Periods × Treatments	ns				***			

DAT: day(s) after treatment, ns: no significant difference

Different letters in each column indicates significant difference at *P* < 0.05 as computed by Tukey’s test. The start marks (***) in table indicate significantly different at *P < 0*.*001*. All data were collected from three consecutive repetitions of the same experiment in a time dependent manner. Values are shown as the mean ± standard error (*n* = 30).

**Fig 1:**
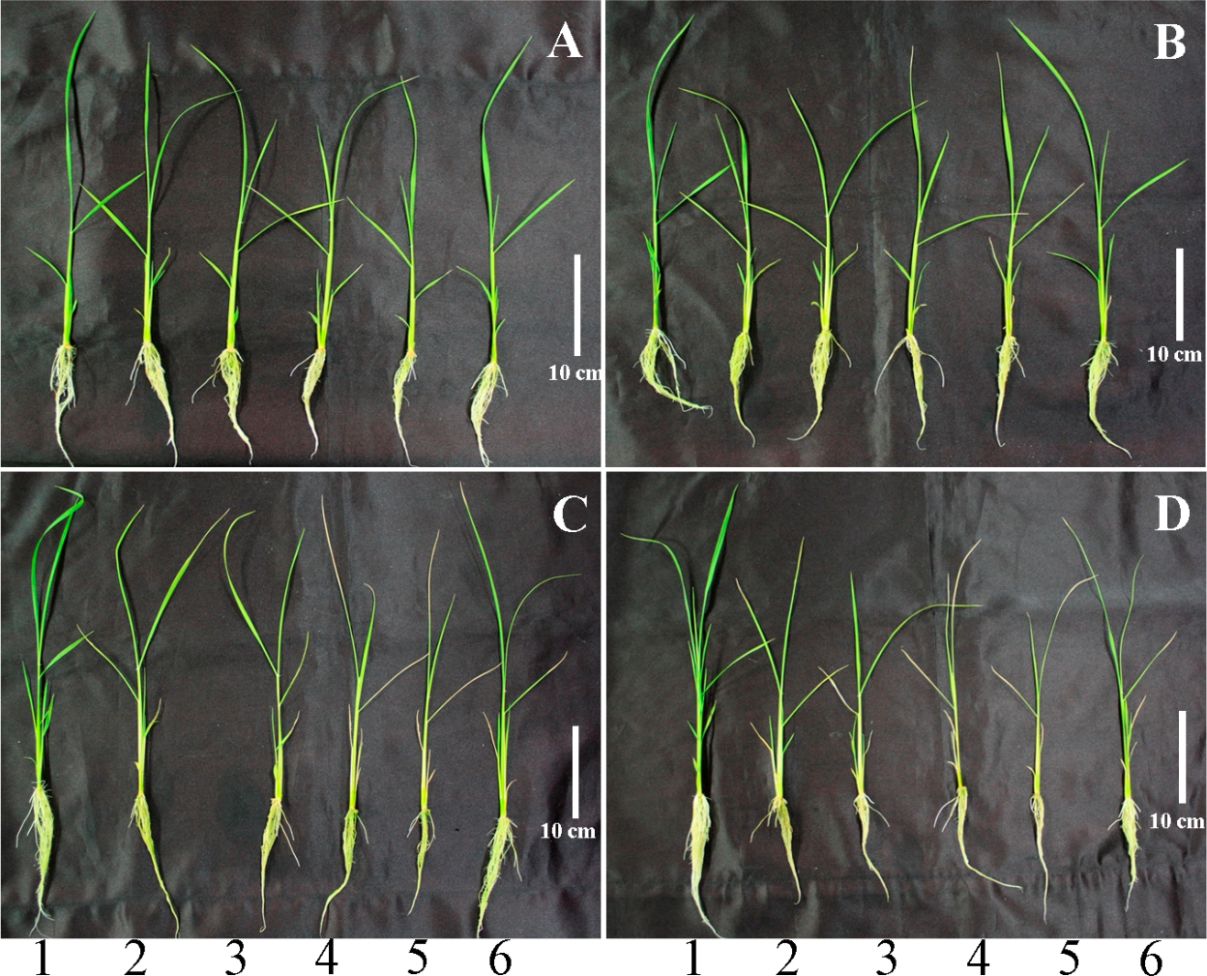
Effect of combined treatment with SA and NaCl for 4 days on phenotypic changes in rice plants. Arabic numbers indicate each treatment (1: control, 2: 0.5 mM SA, 3: 1.0 mM SA, 4: 0.5 mM SA + 100 mM NaCl, 5: 1.0 mM SA + 100 mM NaCl, 6: 100 mM NaCl) and capital letters indicate different treatment periods (A: 1 day, B: 2 days, C: 3 days, D: 4 days). The white vertical bar in the figure represents 10 cm.

The response to SA treatment suggests the presence of a negative feedback loop on growth with or without NaCl as compared to untreated rice plants. When we applied SA with and without NaCl, plant height showed difference among treatments relatively slow because 1DAT to 3DAT did not show any significant difference in plant height (Table 1). However, in case of plant fresh weight, SA or SA with NaCl treated plant showed significant difference from 1DAT as compared to that of control (Table 1). On other hand the phenotypic changes in rice plants following treatment with SA and NaCl alone or in combination are shown in Fig 1. Plant plasticity did not differ at 1 DAT among treatments; however, phenotype results differed significantly from 2–4 DAT. Leaf tips started to undergo necrosis from 2 DAT; the greatest extent of leaf necrosis was observed in rice plants treated with combined SA and NaCl and increased with advancing exposure time (Fig 1B, 1C and 1D). Conversely, in plants treated with NaCl only, leaf necrosis occurred at 3 DAT, and leaf necrosis in SA-only treated plants was observed at 4 DAT (Fig 1C and 1D).

The results showed that sodium ions were almost uniformly distributed in the shoots of rice plants, in both the control and sole SA treated plants, at the different time points. However, the sodium contents declined (NaCl: 29.6%; SA with NaCl: 61.6%) over time when NaCl was applied alone and in combination with SA. However, the decrease in sodium contents were more rapid in SA + NaCl treated plants as compared to SA treated plants (Table 2).

**Table 2:** Sodium ions and SA content of rice seedlings in response to SA application (0.5 and 1.0 mM) in the presence and absence of salt stress (100 mM).

Treatments	Sodium ion content (g kg^-1^ DW)				SA content (μg g^-1^ DW)			
	1DAT	2DAT	3DAT	4DAT	1DAT	2DAT	3DAT	4DAT
Control	1.5 ± 0.03b	1.5 ± 0.07b	1.5 ± 0.05c	1.4 ± 0.03c	1.5 ± 0.15c	1.8 ± 0.01bc	1.6 ± 0.07c	1.1 ± 0.08c
0.5 mM SA	1.4 ± 0.01b	1.5 ± 0.06b	1.5 ± 0.01c	1.4 ± 0.12c	2.9 ± 0.24b	5.5 ± 0.07a	7.2 ± 0.17a	9.1 ± 0.32a
1.0 mM SA	1.4 ± 0.09b	1.5 ± 0.07b	1.5 ± 0.05c	1.3 ± 0.02c	3.1 ± 0.04b	5.8 ± 0.07a	7.8 ± 0.19a	9.6 ± 0.06a
0.5 mM SA +100 mM NaCl	21.1 ± 0.58a	20.4 ± 0.41a	15.0 ± 0.68b	8.1 ± 0.52b	4.8 ± 0.39a	3.6 ± 0.26abc	3.0 ± 0.07b	2.3 ± 0.22b
1.0 mM SA +100 mM NaCl	20.1 ± 0.37a	21.4 ± 0.54a	15.2 ± 0.57b	8.3 ± 0.29b	4.1 ± 1.31ab	3.7 ± 0.08ab	3.0 ± 0.04b	2.2 ± 0.06b
100 mM NaCl	21.6 ± 0.49a	20.7 ± 0.27a	20.4 ± 0.29a	15.2 ± 0.18a	1.0 ± 0.07c	1.4 ± 0.07c	1.6 ± 0.11c	1.7 ± 0.05bc
ANOVA								
Treatment Periods	***				***			
Treatments	***				***			
Replication	ns				ns			
Treatment Periods × Treatments	*				***			

DAT: Day(s) after treatment, SA: salicylic acid

Different letters in each column indicates significant difference at *P* < 0.05 as computed by Tukey’s test. The start marks (*, ***) in table indicate significantly different at *0*.*01 < P < 0*.*05* and *P < 0*.*001* respectively. All data were collected from three consecutive repetitions of the same experiment. Values are shown as the mean ± standard error (*n* = 30).

### Endogenous SA contents in plants treated with NaCl and SA

Endogenous SA contents revealed different phenotypic patterns associated with sodium content (Table 2). At 1 DAT, SA contents were significantly increased (65.52% and 32.26%) in samples treated with SA and NaCl in combination (0.5 mM SA + 100 mM NaCl and 1.0 mM SA + 100 mM NaCl) as compared with the plants treated with SA (0.5 mM and 1.0 mM) respectively. Meanwhile, there were no differences in SA content following treatment with NaCl alone compared with the control. SA content gradually increased as the exposure period advanced in rice treatment with SA or NaCl alone, whereas the SA level gradually decreased with advancing exposure in rice plants treated with SA and NaCl in combination (Table 2).

### Influence of SA and NaCl treatment on chlorophyll florescence and electric conductivity

**Fig 2:**
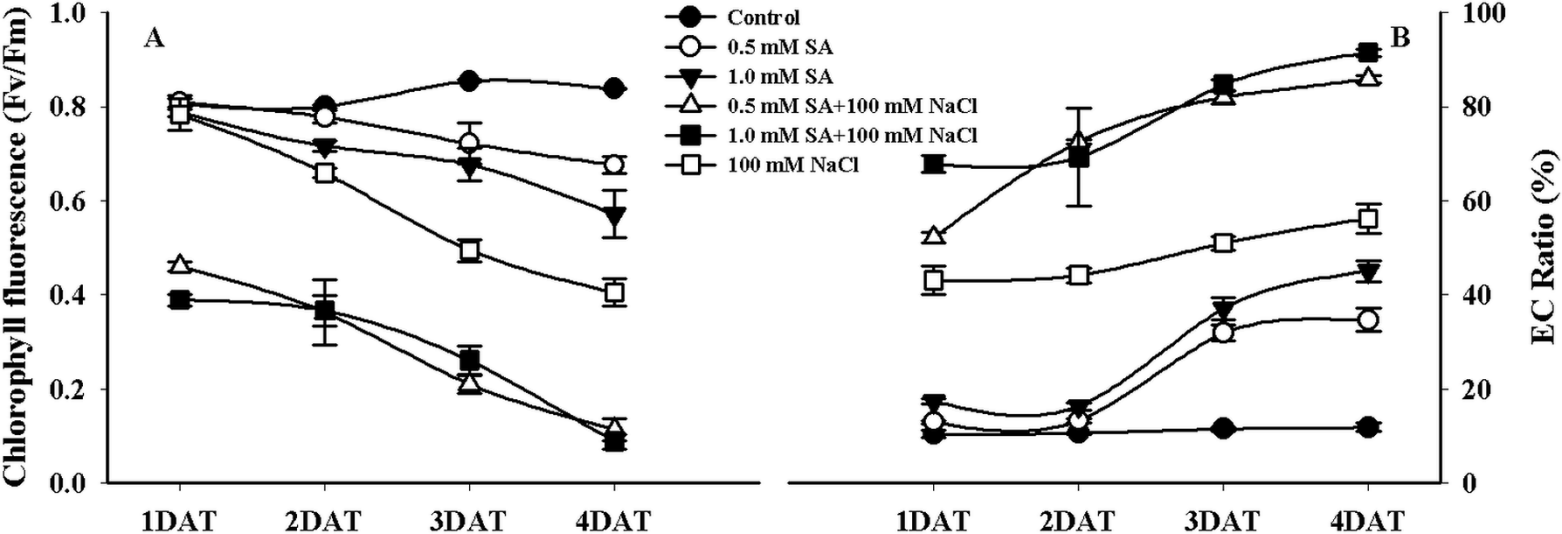
Changes in chlorophyll fluorescence and the electric conductivity ratio after combination treatment of rice plants with SA and NaCl, and single treatment with SA or NaCl. A and B indicate changes in chlorophyll fluorescence and the EC ratio during different treatment times, respectively. Each symbol indicates the average, and the bar indicates ± standard error (n = 3).

The results showed that CF and EC differed among treatments when rice plants were exposed to SA and NaCl alone and in combination (Fig 2). At 1 DAT, the application of SA or NaCl alone resulted in very similar CF levels, whereas CF decreased by approximately 50% at both SA concentrations when combined with NaCl, compared with the control (Fig 2A). Despite increasing the application time, CF values were maintained at a stable level (approximately 0.8 Fv/Fm) in the control plants, whereas CF levels at 2 DAT were slightly decreased under both SA treatments and in the NaCl-only treatment compared to that in the control (Fig 2A). When SA alone or in combination with NaCl was applied for 4 days, CF levels were extremely low; however, at both concentrations of SA with NaCl, and in the NaCl-only treatments, an approximate 50–88.9% decrease occurred relative to that of the control. CF decreased by approximately 10–20% in plants treated with SA alone compared to the control (Fig 2A).

Fig 2 shows that the CF in plants treated with SA and NaCl alone and in combination exhibited a decreasing trend; however, the EC ratio responded differently. In the control, the EC ratio did not significantly change despite an increasing period of stress exposure; however, the EC ratio (30.2–90.2%) increased in rice treated with SA alone and in combination with NaCl with increasing exposure time (Fig 2B). At 1 and 2 DAT, the EC ratio did not differ between both concentrations of SA compared with that of the control; however, the EC ratio significantly increased under both SA conditions (Fig 2B). Conversely, after 1 DAT, the EC ratio differed significantly (52–71%) in rice treated with NaCl alone and in combination with SA compared with the control. Thus, the same tendency was observed in the other treatment periods (2, 3, and 4 DAT) (Fig 2B).

### Changes in hydrogen peroxide contents under single or combined stress conditions

**Fig 3:**
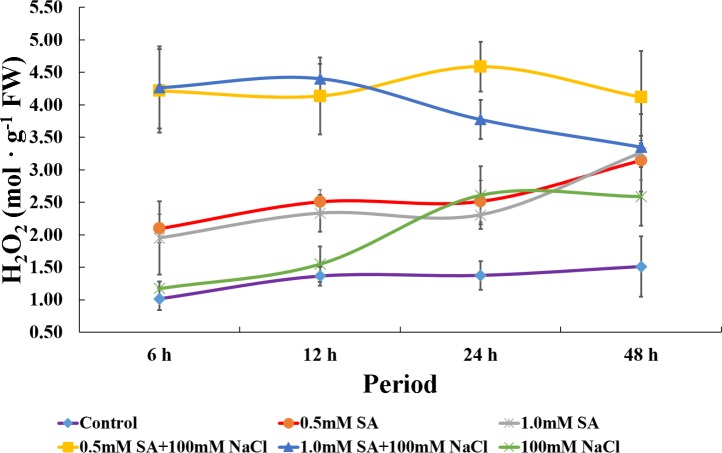
Changes in hydrogen peroxide (H_2_O_2_) contents in rice plants after combination treatment with SA and NaCl, and single treatment with SA or NaCl. Each symbol indicates the average value, and the bar indicates ± standard deviation (n = 5).

To demonstrate ROS generation, we measured hydrogen peroxide (H_2_O_2_) contents in rice plants following stress exposure. Significant accumulations of H_2_O_2_ (approximately 2.0–4.0-fold increase) were observed in rice following treatment with both concentration of SA with NaCl compared with the control, and this tendency was observed until 48-h stress exposure (Fig 3). Rice plants treated with SA only also showed an increased H_2_O_2_ content (approximately 2.0-fold increase) compared with control plants during all time periods, while rice plants treated with NaCl alone showed similar H_2_O_2_ contents to the control plant until 12 h. However, the H_2_O_2_ content was gradually increased after 12-hours stress exposure, with similar H_2_O_2_ levels to the SA-only treated rice plants at 48-hours stress exposure (Fig 3).

### Changes in antioxidant activity after single or combined stress conditions

**Fig 4:**
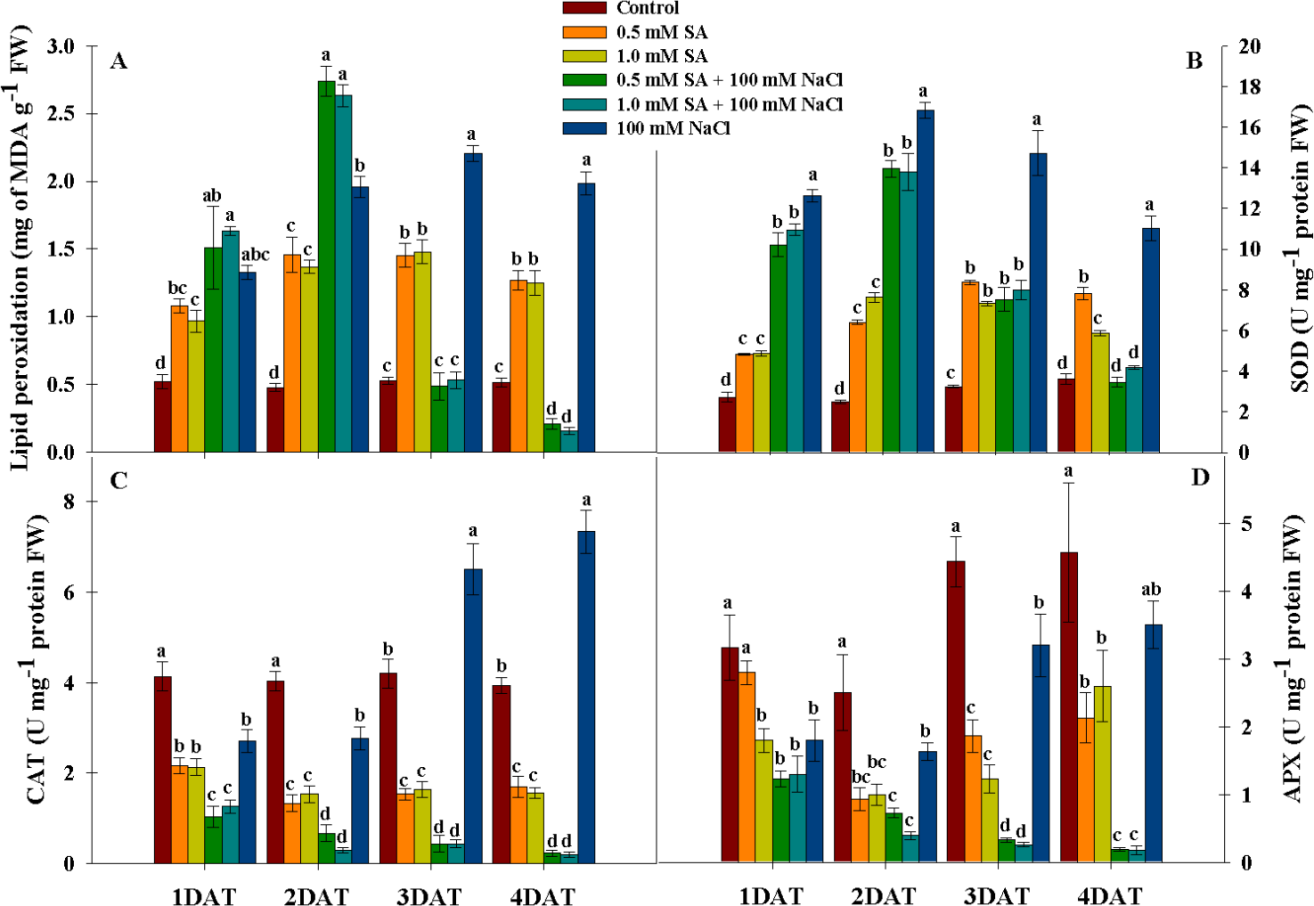
Influence of single application of SA, NaCl, and combination treatment of SA and NaCl on antioxidant activity in rice plants. Uppercase letters indicate each antioxidant, and lowercase letters show significant differences among treatments within the same treatment period. Different letters indicate significance difference at P < 0.05, and data were analyzed by Duncan’s multiple range test (DMRT). DAT = day(s) after treatment.

To confirm stress induction, we assessed the antioxidant activity of stress-exposed plants. Lipid peroxidase activity (MDA) was significantly increased (approximately 2.0–3.0-fold increase) under all treatments compared to that in the control at 1 DAT (Fig 4A). Particularly, MDA activity at 1 DAT was highest in plants exposed to both concentrations of SA with NaCl, second highest in those exposed to NaCl only, followed by the 0.5 and 1.0 mM SA only applications. At 2 DAT, MDA activity exhibited the same pattern observed at 1 DAT, whereas MDA activity exhibited a different tendency at 3 and 4 DAT. At 3 DAT, MDA activity was almost the same between plants treated with SA in combination with NaCl and the control. At 4 DAT, MDA activity reduced (45–51%) to a low level compared with that observed at other time points (Fig 4A).

SOD activity was markedly increased (approximately 2.0–8.0-fold increase) in plants treated with SA or NaCl only, and SA with NaCl in combination, compared with the control, and this tendency continued at 3 DAT (Fig 4B). However, SOD activity varied at 4 DAT. SOD activity remained higher in the SA or NaCl-only treatments than in the control; however, SOD activity in plants treated with SA and NaCl in combination did not differ significantly compared with the control (Fig 4B).

The activity of CAT is shown in Fig 4C. At 1 DAT, CAT exhibited higher activity in the control than in the other treatments, and this was also observed at 2 DAT. However, CAT activity differed between plants at 3 and 4 DAT. NaCl treatment resulted in the highest CAT activity at 3 and 4 DAT compared with the other treatments (Fig 4C). From 3 DAT, CAT activity remained significantly higher (33.3–43.0%) in the NaCl-only treatment compared with the control. Conversely, CAT activity decreased in rice plants treated with SA alone and in combination with NaCl compared with the control. The lowest level of CAT activity was observed in plants treated with SA and NaCl in combination (Fig 4C). Consequently, there was a decrease in CAT activity with SA treatment. APX activity exhibited the same tendency as CAT activity and was significantly lower in treated plants at all exposure periods compared with the control (Fig 4D). Particularly, APX activity was significantly lower in plants treated with the combination of SA and NaCl compared with those treated with SA or NaCl alone (Fig 4D).

### Expression of the antioxidant genes *OsCATA* and *OsAPX1*

**Fig 5:**
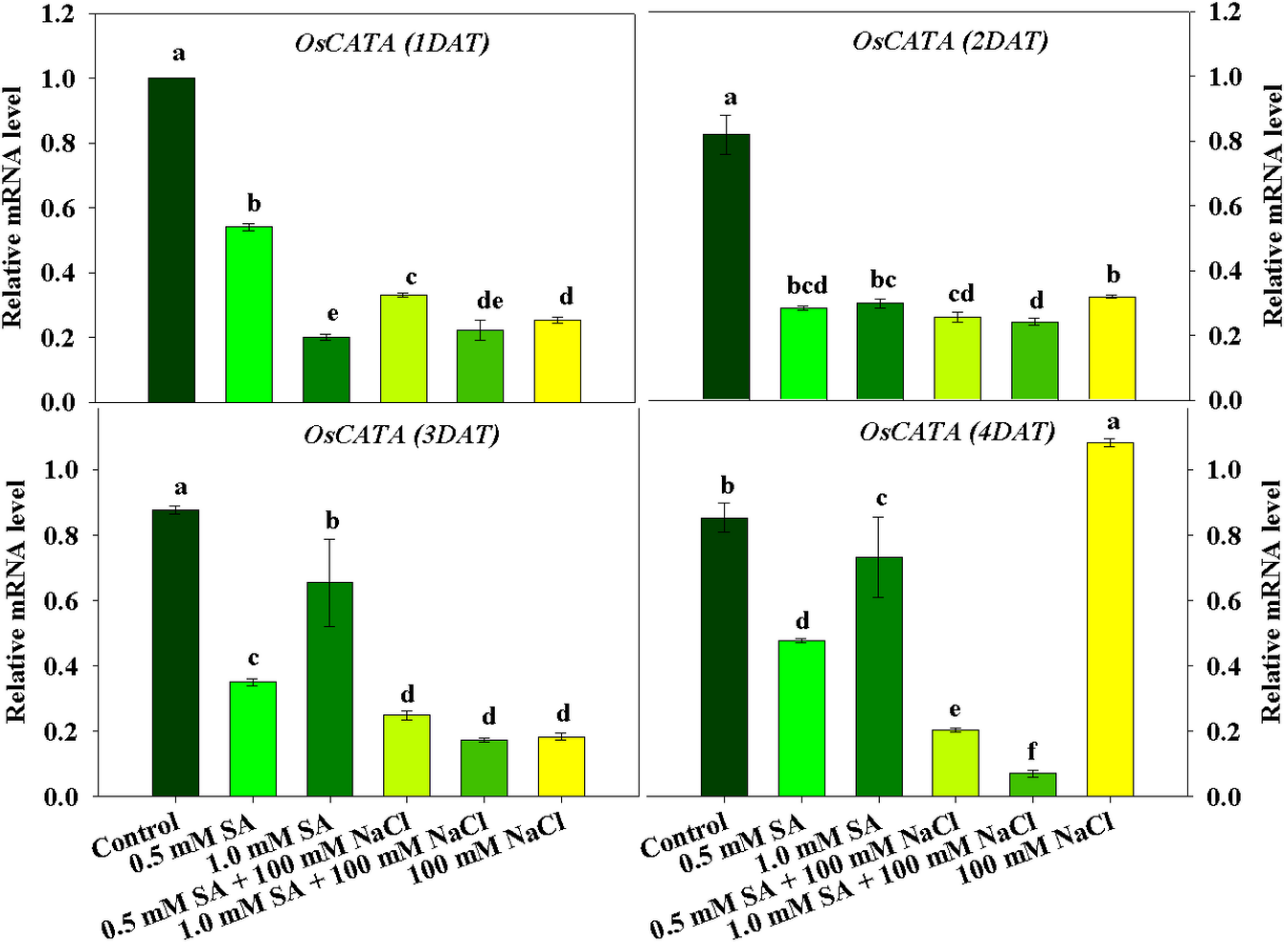
Effects of single application of SA or NaCl, and combination treatment of SA and NaCl on *OsCATA* expression in rice plants. Lowercase letters indicate a significant difference among treatments. Vertical bars with error bars indicate the average ± standard error (n = 3), and different letters indicate a significance difference at P < 0.05. All data were analyzed by DMRT.

**Fig 6:**
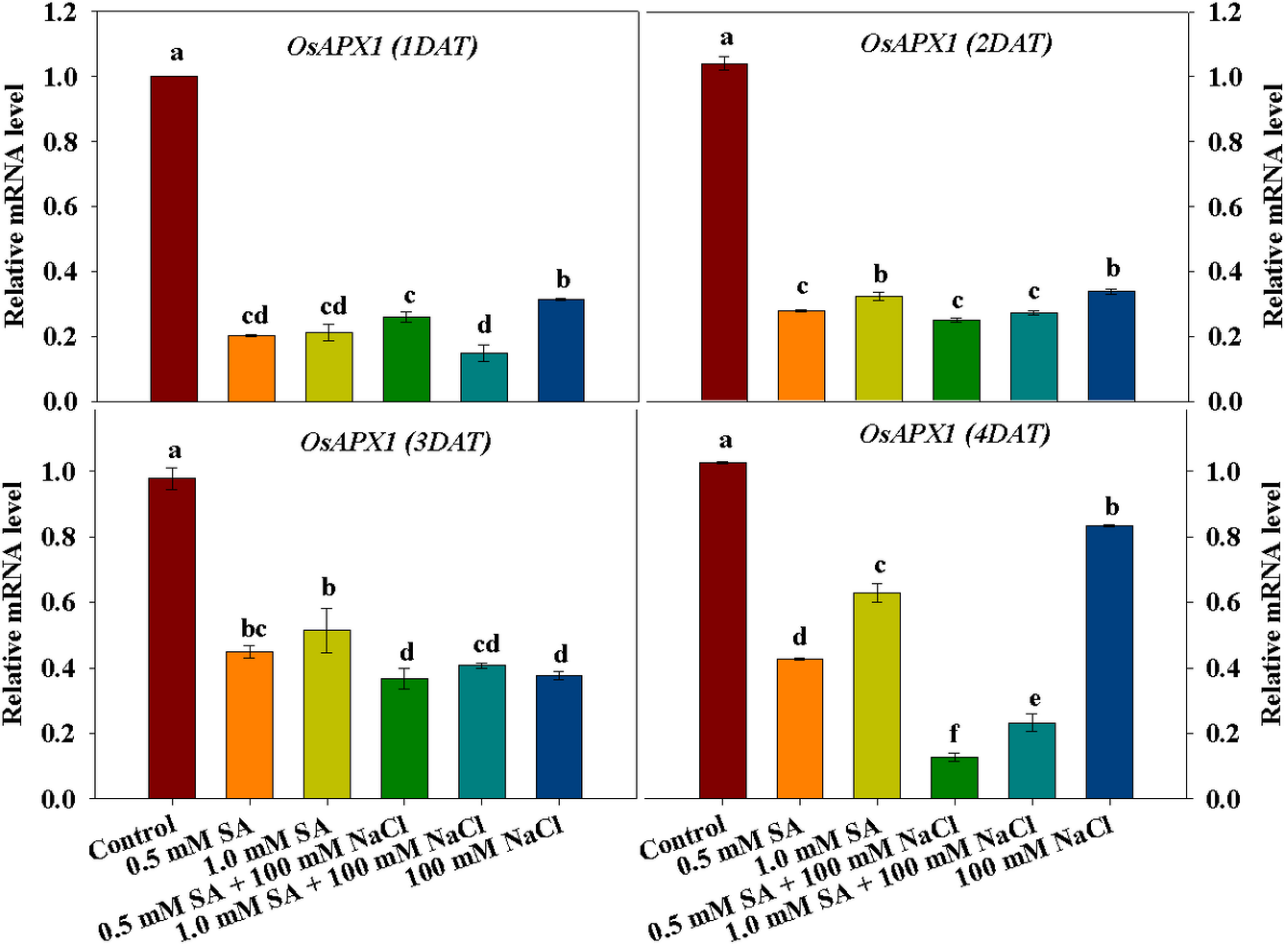
Effects of single application of SA or NaCl, and combination treatment of SA and NaCl on *OsAPX1* expression in rice plants. Lowercase letters indicate a significant difference among treatments. Vertical bars with error bars indicate the average ± standard error (n = 3), and different letters indicate significant differences at P < 0.05. All data were analyzed by DMRT.

To identify changes in antioxidant activity at the molecular level, we assessed the mRNA expression levels of *OsCATA* and *OsAPX1*. The results are illustrated in Figs 5 and 6. As shown in Fig 5, mRNA expression of *OsCATA* was down-regulated in plants treated with all SA and NaCl treatments at 1 and 2 DAT (Fig 5). However, the expression of these genes increased slightly from 3 DAT in plants treated with SA or NaCl only, whereas mRNA expression of *OsCATA* remained low in plants treated with combined SA and NaCl (Fig 5C and 5D). The mRNA expression of *OsAPX1* followed a similar tendency to that of *OsCATA*. There was a significant decrease in the mRNA expression of *OsAPX1* in plants treated with SA and NaCl alone and in combination, compared with the control. Notably, the lowest expression of *OsAPX1* was observed following treatment with combined SA and NaCl at 4 DAT (Fig 6).

### Pearson’s correlation coefficient among phenotypic data

To identify correlations between phenotypic data (shoot length, fresh weight, chlorophyll fluorescence), Fv/Fm, and antioxidant activity, we conducted a correlation test using all phenotype data for each exposure period. There was no significant correlation between shoot length and antioxidant activity, whereas a strong correlation was observed for biomass with CAT (2 and 3 DAT) and APX (3 and 4 DAT) (Table 3). Fv/Fm was significantly positively correlated with the activity of CAT and APX at 1 and 2 DAT; however, only APX activity was consistently significantly correlated (r = 0.592–0.753) with Fv/Fm (Table 3). Antagonistic results of Fv/Fm were observed in EC for the shoot area of rice. Significant negative correlations between EC, and CAT and APX activity were investigated at all time points (except for CAT at 4 DAT), whilst consistent positive correlations were observed between EC and MDA and SOD activity at 1 and 2 DAT (Table 3). Comparison among phenotypic results (SL, FW, Fv/Fm, and EC) indicated that SL was positively correlated with FW at 3 and 4 DAT; however, FW revealed a significant positive correlation with Fv/Fm and a significant negative correlation with EC at 3 and 4 DAT (Table 3).

**Table 3:** Pearson’s correlation coefficient among phenotypic data in different stress exposure periods of rice plant.

		CAT	MDA	SOD	APX	Fv/Fm	EC	SL	FW
1DAT	CAT	-	-0.791**	-0.449ns	0.773**	0.701**	-0.664**	-0.224ns	0.387ns
	MDA		-	0.690**	-0.656**	-0.698**	0.799**	-0.063ns	-0.271ns
	SOD			-	-0.652**	-0.608**	0.884**	-0.088ns	-0.088ns
	APX				-	0.650**	-0.774**	-0.300ns	0.197ns
	Fv/Fm					-	-0.866**	-0.206ns	0.113ns
	EC						-	0.108ns	-0.097ns
	SL							-	0.205ns
	FW								-
2DAT	CAT	-	-0.782**	-0.306ns	0.842**	0.694**	-0.592*	0.302ns	0.587*
	MDA		-	0.787**	-0.710**	-0.865**	0.908**	0.014ns	-0.418ns
	SOD			-	-0.279ns	-0.706**	0.859**	0.211ns	-0.120ns
	APX				-	0.592*	-0.506*	0.437ns	0.390ns
	Fv/Fm					-	-0.905**	-0.091ns	0.288ns
	EC						-	0.225ns	-0.246ns
	SL							-	0.386ns
	FW								-
3DAT	CAT	-	0.600**	0.513*	0.822**	0.405ns	-0.472*	0.211ns	0.728**
	MDA		-	0.444ns	0.276ns	0.267ns	-0.261ns	-0.231ns	0.061ns
	SOD			-	0.043ns	-0.529*	0.481*	0.032ns	0.095ns
	APX				-	0.721**	-0.779**	0.265ns	0.818**
	Fv/Fm					-	-0.970**	0.132ns	0.508*
	EC						-	-0.095ns	-0.594**
	SL							-	0.574*
	FW								-
4DAT	CAT	-	0.734**	0.850**	0.692**	0.384ns	-0.442ns	0.221ns	0.434ns
	MDA		-	0.724**	0.505*	0.373ns	-0.369ns	-0.239ns	-0.080ns
	SOD			-	0.321ns	-0.092ns	0.040ns	-0.057ns	-0.014ns
	APX				-	0.753**	-0.820**	0.339ns	0.723**
	Fv/Fm					-	-0.980**	0.288ns	0.631**
	EC						-	-0.325ns	-0.697**
	SL							-	0.683**
	FW								-

In the table, asterisks (*) indicate Pearson’s correlation coefficient (*r*) among phenotypic data and each start mark showed significantly different at *P* < 0.01 (**) or *P* < 0.05 (*). In the table, ns mean no difference between data and DAT in left side of table indicated day after treatment (stress exposure period).

### SNO contents and (S)NO related genes expression

**Fig 7:**
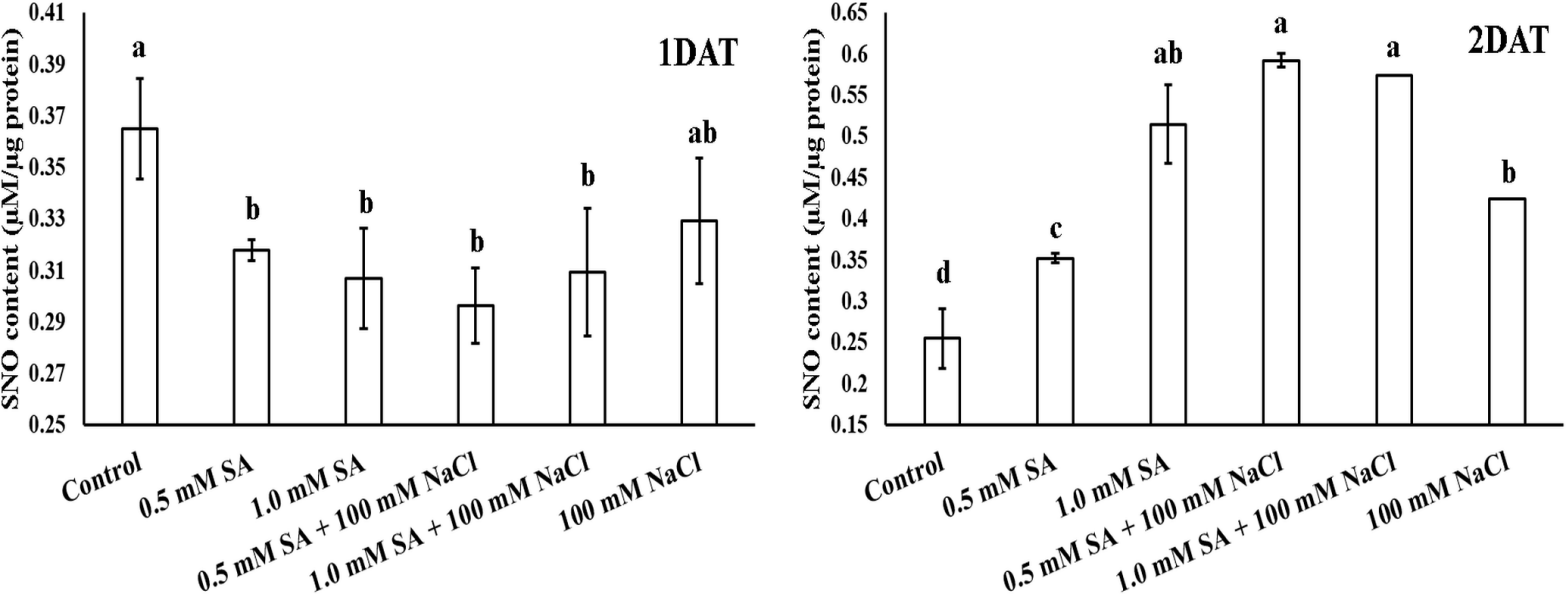
Effects of single application of SA or NaCl, and combination treatment of SA and NaCl on SNO contents in rice plant. Lowercase letters indicate a significant difference among treatments. DAT (days after treatment) refers to the period of SA and NaCl exposure. Vertical bars with error bars indicate the average ± standard error (n = 3), and different letters indicate a significant difference at P < 0.05. All data were analyzed by DMRT.

To predict NO scavenging activity, we analyzed SNO contents and the expression levels of related genes, such as *OsGSNOR*, *OsNOA*, and *OsNR* at one-day intervals for 2 days. Most SA and NaCl treatments resulted in a significant decrease in SNO contents compared with the control at 1 DAT; however, a markedly different pattern was exhibited at 2 DAT (Fig 7). Application of SA and NaCl alone and in combination resulted in a significant increase in SNO contents compared with the control (Fig 7). Particularly, SNO contents were most increased in plants treated with SA and NaCl in combination compared with those treated with SA or NaCl alone (Fig 7).

**Fig 8:**
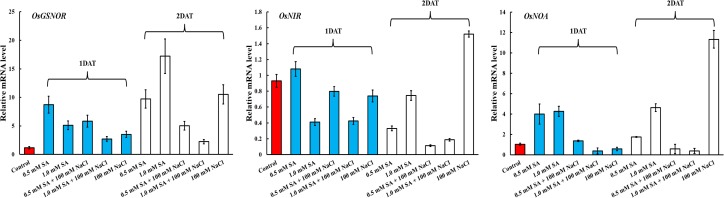
Effects of single application of SA or NaCl, and combined treatment of SA and NaCl on the mRNA expression of *OsGSNOR*, *OsNIR*, and *OsNOA* in rice plants. Lowercase letters indicate a significant difference among treatments. DAT (days after treatment) refers to the period of SA and NaCl exposure. Vertical bars with error bars indicate the average ± standard error (n = 3), and different letters indicate a significant difference at P < 0.05. All data were analyzed by DMRT.

*OsGSNOR* encodes an NO scavenger; therefore, we measured the mRNA expression of this gene. Overall, the mRNA expression of *OsGSNOR* increased in plants treated with SA and NaCl alone and in combination (Fig 8). Comparisons between plants treated with SA alone and in combination with NaCl revealed that *OsGSNOR* expression was higher in SA-only treated rice plants than in those treated with SA and NaCl at 1 and 2 DAT (Fig 8). Conversely, *OsNR* expression decreased in plants treated with SA alone and in those treated with SA in combination with NaCl compared with the control at 1 DAT (Fig 8). At 2 DAT, only plants treated with SA alone and SA in combination with NaCl revealed a decreased expression of this gene compared with the control; however, only NaCl-treated rice plants exhibited increased mRNA expression levels compared with the control plants (Fig 7). Increased mRNA expression of *OsNOA* was observed in rice plants treated with SA alone compared with the control at 1 and 2 DAT, while increased *OsNOA* expression was found only at 2 DAT (Fig 8). Rice plants treated with combined SA and NaCl exhibited similar or decreased *OsNOA* expression compared with the control plant during all experimental periods.

## Discussion

With the current changes in global climatic conditions, the occurrence of multiple stress conditions (biotic and abiotic) can be detrimental to crop growth and production [44]. Over the past few decades, SA (endogenous or exogenous) has been identified as a key regulator of physiological, biochemical, and genetic mechanisms against various stress conditions, such as pathogen or insect attacks and abiotic stresses in plants [45, 46]. However, high concentrations of exogenously applied SA can induce artificial biotic stress in plants and can inhibit the ROS scavenging pathway in plants; elevated levels of SA inhibit the activity of APX and CAT [30]. Furthermore, elevated levels of SA in plants markedly enhance plant cell death due to increased production of H_2_O_2_ and lipid peroxidation [47, 48]. Conventionally, exogenous application of NaCl has been used to induce artificial salinity stress in many studies; since NaCl application induces osmotic imbalance in the cell, we applied NaCl to induce abiotic stress (salinity stress) [2, 49]. The results of the current study support the negative effects of SA on rice plant growth. The combination of biotic (SA, 0.5 and 1.0 mM) and abiotic (NaCl) stress led to changes in plant plasticity by significantly affecting the leaf length and biomass. In addition, the sodium ion content differed between plants treated with NaCl and SA.

The results showed that SA and NaCl markedly influence the photosynthetic efficiency of rice plants. Overall, the CF tended to decline, except in control plants. Particularly, the CF was significantly lower in plants treated with SA, at both concentrations, in combination with NaCl compared with the NaCl-only treatments; however, the result for the EC ratio differed. The EC significantly increased in plants treated with SA compared with the control. The high EC ratio indicated the possibility of damage to the photosynthesis apparatus, chloroplast, or damage to the cell membrane. Photosynthesis is separated into two phases, photosystem I (PSI) and photosystem II (PSII), and CF is known to reflect PSII photochemicals in dark-adapted leaves [50]. If plants are exposed to stresses induced by biotic or abiotic factors, the photosynthetic apparatus is damaged, which could affect the CF values, a physiological indicator of stress [50, 51]. Poór et al. [34] applied 10^−3^ M SA and NaCl to tomato plants and induced PCD and electrolyte leakage in response to accelerated ROS accumulation. Thus, in the present study, ROS and related scavenging enzymatic activity were assessed to provide an in-depth understanding of the physiological, biochemical, and genetic changes in rice plants.

Antioxidants counteract oxidative stresses in plant cells and contribute to the maintenance of homeostasis under oxidative stress [52]. The results of the present study revealed various levels of antioxidant activity in plants treated with SA and NaCl alone and in combination. Lipid peroxidation (a measure of oxidative stress to the cell lipid bi-layer), exhibited high MDA activity in response to combined SA and NaCl application compared with other treatments until 2 DAT. MDA activity was markedly decreased in plants treated with combined SA and NaCl at 3 and 4 DAT. A similar pattern was observed for SOD activity. MDA and SOD exhibited higher activity in plants treated with SA only and in combination with NaCl compared with the control; however, antioxidant activity declined after 3 DAT. These results support the phenotypic changes induced by SA with NaCl treatments, with accelerated leaf discoloration in rice plants after 3 DAT suggesting accelerated plant cell death. García-Heredia et al. [30] and Poór et al. [34] showed that SA and ASA accelerated PCD in *Arabidopsis*, as indicated by typical PCD-linked morphological and biochemical changes. In the present study, application of SA at 0.5 and 1.0 mM induced oxidative stress.

SOD may function to scavenge O_2_^-^ and H_2_O_2_ to activate other defense mechanisms [53, 54]. Interestingly, CAT and APX activity was downregulated in plants treated with combined SA and NaCl. Therefore, artificial abiotic and biotic stress conditions can inhibit the activation of CAT and APX and thus, lead to the accumulation of H_2_O_2_ in plant cells.

In general, antioxidants participate in ROS scavenging to mitigate environmental stresses; therefore, the induction of phenotypic changes is possible through the activity of antioxidants [52]. Similar results have been reported in several studies. According to Motamedi and Farhoudi [55], different levels of salinity (4, 6, 8, 10, and 12 dS/m) in safflower resulted in a strong correlation between the activity of several antioxidants and shoot and root length. Seedling fresh weight was also significantly positively correlated with CAT and POD activity [55]. In contrast, our results indicated that antioxidant activity (CAT, MDA, SOD, and APX) was not a good predictor of plant length, while CAT and APX activity could be used to predict plant biomass following the exposure to plants to environmental stress conditions.

In addition to other oxidants, NO is involved in plant physiological processes, such as germination, leaf expansion, hormone regulation, and response to stress conditions [13–15, 56]. The key role of NO is to induce plant immune responses, such as compromised SA, reduced hypersensitive response (HR), and disease susceptibility [15]. According to Zurbriggen et al. [57], ROS are also involved in HR or localized cell death (LCD) during pathogen invasion. Therefore, the balance of ROS and NO in plant cells is an essential strategy to avoid or mitigate stress conditions [56]. Here, we measured the expression of SNO- and (S)NO-related genes to elucidate crosstalk between antioxidants and NO scavengers or producers. Our results showed that SNO contents in plants were decreased in response to all treatments compared with the control; however, SNO contents were markedly increased in response to all treatments at 2 DAT. SNO contents were particularly increased in plants treated with SA in combination with NaCl compared to those treated with SA only. *GSNOR* is a key, indirect regulator of SNO in plants [56]. Therefore, to elucidate the SNO response on a genetic level, we analyzed the mRNA expression of *OsGSNOR*. The data showed that *OsGSNOR* expression was higher in response to all treatments compared to the control, and this tendency was observed from 1–2 DAT. Only SA-treated rice plants exhibited higher *OsGSNOR* expression than SA with NaCl-treated rice plants. At 1 DAT, despite the decrease in SNO contents observed in response to all treatments, the expression of *OsGSNOR* increased compared to the control. It is possible that these results reflect differences in signal reaction. *OsNR* is involved in the reduction of nitrate to nitrite; therefore, enhanced *OsNR* expression participates in NO generation in plants [58–59]. According to our results, *OsNR* expression gradually decreased with increasing exposure period in rice plants treated with SA alone and in combination with NaCl compared to the control plants.

## Conclusions

Studies on the physiological responses of plants exposed to multiple stress conditions are limited; thus, our study involved the application of SA and NaCl to rice plants to induce artificial abiotic and biotic stress. Our results showed that cell death was induced in rice plants treated with combined SA and NaCl through the downregulation of antioxidant activity of CAT as well as APX. The activity of these enzymes was regulated at the genetic level. Finally, artificial abiotic and biotic stress conditions induced the downregulation of *OsCATA* and *OsAPX1*, resulting in reduced enzyme activity (CAT and APX). Under stress conditions (single and multiple stress conditions), rice growth characteristics were significantly correlated with CAT and its mRNA expression level; therefore, we assume that CAT activity was most sensitive to stress. Both stress conditions could induce the upregulation of *OsGSNOR* and, thus, increase SNO contents.

## Supporting information

S1 Table HPLC conditions for SA analysis. (DOCX)

S2 Table The primers used for real-time PCR. (DOCX)
