# Supplementary material for: Regulation of reactive oxygen and nitrogen species by salicylic acid in rice plants under salinity stress conditions
Source: PLoS One. 2018 Mar 20;13(3):e0192650. doi: 10.1371/journal.pone.0192650 (PMC5860692; doi:10.1371/journal.pone.0192650)
Supplement: S1 Table — (DOCX) [file pone.0192650.s001.docx]

**S1 Table.** HPLC conditions for SA analysis

| HPLC conditions for SA analysis | |
| --- | --- |
| Equipment | Shimadzu couple with RF-10AXL fluorescence detector |
| Column | C18 reverse-phase (HP hypersil ODS; particle size = 5 mM; pore size = 120-A° water) |
| Wavelength | 305-365 nm |
| Flow rate | 1.0 ml min^-1^ |
| Solvent A, B | Solvent A: 100% MeOH, Solvent B: 100% water in 0.5% acetic acid |
| Gradient  (A% / B%) | (30/70) 5 min → (40/60) 2.5 min → (60/40) 4.5 min → (30/70) 5 min → (30/70) 3 min |
