# Supplementary material for: Regulation of reactive oxygen and nitrogen species by salicylic acid in rice plants under salinity stress conditions
Source: PLoS One. 2018 Mar 20;13(3):e0192650. doi: 10.1371/journal.pone.0192650 (PMC5860692; doi:10.1371/journal.pone.0192650)
Supplement: S2 Table — (DOCX) [file pone.0192650.s002.docx]

**S2 Table. The primers used for real-time PCR.**

| Gene | Primer sequences |
| --- | --- |
| *OsUBI* | Forward: GACGGACGCACCCTGGCTGACTAC  Reverse: TGCTGCCAATTACCATATACCACGAC |
| *OsAPX1* | Forward: CCAAGGGTTCTGACCACCTA  Reverse: CAAGGTCCCTCAAAACCAGA |
| *OsCATA* | Forward: CGGATAGACAGGAGAGGTTCA  Reverse: AATCTTCACCCCCAACGACT |
